# Major *LOXL1* risk allele is reversed in exfoliation glaucoma in a black South African population

**Published:** 2010-04-21

**Authors:** Susan E.I. Williams, Benjamin T. Whigham, Yutao Liu, Trevor R. Carmichael, Xuejun Qin, Silke Schmidt, Michele Ramsay, Michael A. Hauser, R. Rand Allingham

**Affiliations:** 1 Division of Ophthalmology, Department of Neurosciences, University of the Witwatersrand, Johannesburg, South Africa; 2 Center for Human Genetics, Duke University Medical Center, Durham, NC; 3 Division of Human Genetics, NHLS and School of Pathology, University of the Witwatersrand, Johannesburg, South Africa; 4 Department of Ophthalmology, Duke University Eye Center, Durham, NC

## Abstract

**Purpose:**

To investigate whether variants in the lysyl oxidase-like 1 (*LOXL1*) gene are associated with exfoliation glaucoma (XFG) and primary open-angle glaucoma (POAG) in an ancestral population from South Africa.

**Methods:**

Black South African subjects with XFG, POAG, and age matched unaffected controls were recruited from the St. John Eye Hospital in Soweto, Johannesburg, South Africa, using standard clinical examination techniques. Fifty individuals were collected for each of the three groups: XFG, POAG, and normal controls. The complete coding region of *LOXL1* was sequenced using the PCR-based Sanger method. The allele frequencies of the identified sequence variants were compared between XFG or POAG and controls using Fisher’s exact test.

**Results:**

A large number of coding variants were identified, including rs1048661 (R141L), rs3825942 (G153D), S159A, S161L, rs41435250 (A320A), rs13329473 (F489F), and T567A. The allele frequencies of both rs3825942 and rs1048661 differed significantly between the XFG and control subjects from South Africa (p=5.2×10^−13^ and 1.7×10^−5^, respectively). The G allele for rs1048661 (encoding arginine) was the risk allele which is similar to other populations. The A allele of rs3825942 (encoding aspartic acid) was the risk allele, in sharp contrast to the G allele (encoding glycine) reported in multiple other populations. There was no significant difference in the allele frequencies of coding variants in *LOXL1* between POAG and control subjects.

**Conclusions:**

This represents the first genetic association study of *LOXL1* in an ancestral African population with XFG. We have confirmed the association between variants of *LOXL1* and XFG. To date, the G allele of the major susceptibility variant rs3825942 has consistently been shown in multiple populations to increase the risk of XFG. Surprisingly, we have found a strong association with the opposite allele in the South African population. This suggests that other as yet unknown causal variants of *LOXL1* contribute to the genetic risk of XFG.

## Introduction

Glaucoma is a heterogeneous group of disorders that are defined by a shared characteristic of progressive loss of retinal ganglion cells, optic nerve cupping, and visual field loss. Glaucoma is the most common cause of irreversible blindness worldwide and primary open-angle glaucoma (POAG) is the single most common type [1]. POAG is a particular problem in people of African descent where it is more common, occurs at a younger age and progresses more rapidly [2-4]. Exfoliation glaucoma (XFG) is the most common identifiable cause of open-angle glaucoma [5]. XFG occurs in the context of exfoliation syndrome (XFS), a systemic condition characterized by pathological deposits of microfibrillar material within the anterior segment of the eye as well as in various extraocular tissues [6]. The prevalence of XFS and XFG varies widely in different populations. XFS is most common in Greek [7] and Nordic populations with a prevalence of over 10% in Iceland that increases with age [8]. It is uncommon in African Americans [9,10], and virtually non-existent in West Africa [11]. However, XFG is the cause of approximately 16 to 20% of glaucoma in black South Africans [12,13]. This ancestral population of speakers of southern Bantu languages can be subdivided into three distinct linguistic groups (Nguni, Sotho/Tswana, and Venda), but they are relatively similar genetically [14].

In 2007 Thorleifsson et al. [15],  in their genome-wide association study, found a strong association between XFG/XFS and common sequence variants in the lysyl oxidase-like 1 (*LOXL1)* gene on chromosome 15q24.1. Three significantly associated single nucleotide polymorphisms (SNPs) were identified: two nonsynonymous coding SNPs, rs1048661 and rs3825942, located in exon 1 of *LOXL1,* and an intronic SNP, rs2165241, located in intron 1 [15]. These findings have subsequently been replicated in numerous populations globally [16-34], but to date there has not been a study of *LOXL1* in an ancestral African population with XFG or XFS. Of the reported non-synonymous *LOXL1* risk variants only rs3825942, which codes for an amino acid change from glycine to aspartic acid at position 153 (G153D), has been consistently associated with XFS and XFG [35]. Interestingly, this risk variant is the common variant in all populations studied to date and has an allele frequency that ranges between 80 and 90% in non-African populations [35]. In contrast, opposite alleles at the rs1048661 coding change have been found to be associated with an increased XFG risk in different populations [35]. The purpose of this study was to examine the role of *LOXL1* sequence variants in a black South African population with XFG and POAG.

## Methods

### Study participants

This study adhered to the tenets of the Declaration of Helsinki. The research protocol was approved by the University of the Witwatersrand Human Research Ethics Committee (Johannesburg, South Africa; protocol number M080817). Southern African black participants with clinically diagnosed XFG or POAG and unaffected southern African control subjects were recruited from the St. John Eye Hospital in Soweto, Johannesburg, South Africa. Written informed consent was obtained from all participants. The home language of participants and that of their parents and grandparents was used to establish their ethnic affiliation. All participants underwent a standardized detailed ophthalmic examination by the same ophthalmologist (S.E.I.W.). The examination included measurement of intraocular pressure (IOP) by applanation, slit lamp biomicroscopy, gonioscopy, and dilated pupil examination of the lens and fundus. Subjects with XFG were defined as those with clinical evidence of exfoliation material on the pupil margin, anterior lens surface, and the presence of glaucomatous optic neuropathy and visual field loss. Subjects with POAG had evidence of glaucomatous optic neuropathy and visual field loss with open angles on gonioscopy and no evidence for a secondary cause for the glaucoma and no clinical evidence of exfoliation. Southern African subjects with normal anterior segment and optic nerve examination, an IOP of less than 18 mmHg and without clinical signs of exfoliation were recruited as control subjects.

### DNA analysis

Genomic DNA was extracted using a salting out procedure from nucleated cells from the venous blood samples of all subjects [36]. Primers flanking the entire coding sequence of *LOXL1* were either designed with Primer3 software [37] or as reported by Fan et al. [34]. Primer sequences are provided in Table 1. A pair of primers was also designed to sequence a potential promoter variant rs16958477 based on a recent study [38]. All the primers were designed to cover at least 30 base pairs into the intronic region to cover potential sequence variants affecting exon splicing. All sequencing was performed using appropriately selected primers and conditions optimized in a standard fashion. Platinum Taq DNA polymerase (Invitrogen, Carlsbad, CA) was used for all the PCR reactions. The PCR reactions were performed in ThermoHybaid MBS 02, 02S, and 02G PCR machines (Thermo Scientific, Waltham, MA). PCR 1d was run with a three-stage program (94 °C for 3 min; then 94 °C for 5 s, 61 °C for 30 s, 72 °C for 45 s over 40 cycles, then 72 °C for 6 min over 1 cycle). The PCR program for sequencing rs16958477 was similar but with a 60 °C annealing step and only over 35 cycles. The other PCR reactions were performed using a touchdown program (94 °C for 3 min; then 94 °C for 5 s, 65 °C for 30 s, 72 °C for 1 min over 2 cycles; 94 °C for 5 s, 63 °C for 30 s, 72 °C for 1 min over 2 cycles; 94 °C for 5 s, 61 °C for 30 s, 72 °C for 1 min over 2 cycles; 94 °C for 5 s, 59 °C for 30 s, 72 °C for 1 min over 2 cycles; 94 °C for 5 s, 57 °C for 30 s, 72 °C for 1 min over 2 cycles; 94 °C for 5 s, 55 °C for 30 s, 72 °C for 1 min over 30 cycles; and 72 °C for 3 min). The final annealing temperature was raised to 56 °C for the 1c1, 1c2, and 1e primer reactions. Mg^2+^ concentration was 2.0 mM for exon 7 PCR and 1.5 mM for all other reactions. Sequencing reactions were performed using BigDye® Terminator v3.1 Cycle Sequencing Kits and run on the ABI 3730 DNA analyzer (Applied Biosystems, Foster City, CA). All the sequence analysis was done by using the Sequencher 4.9 software package (Gene Codes, Ann Arbor, MI). Allele frequencies at rs1048661 and rs3825942 were confirmed by bi-directional sequencing with two sets of primers (1c1 and 1c2).

**Table 1:** List of PCR primers for *LOXL1* (lysyl oxidase-like 1) exon sequencing in South African black individuals with or without exfoliation glaucoma.

***LOXL1* exon**	**Forward primer sequence**	**Reverse primer sequence**	**PCR product size (bp)**	Covered genomic region*
Promoter	CCACCAACAAAGAGGGTGTG	ACCGCCTGTGGGCCTTAC	597	chr15:72,005,330–72,005,926
Exon1a	TCCCAGCCTGTTGCTTATTC	AGGCCTGGTGGACAGAGAG	326	chr15:72,005,836–72,006,161
Exon1b	AAGCAAGGAGCCTTCCTGTC	GCACCCGGGAGCTACTCT	330	chr15:72,006,074–72,006,403
Exon1c1	GCAGGTGTACAGCTTGCTCA	ACACGAAACCCTGGTCGTAG	464	chr15:72,006,324–72,006,787
Exon1c2	GCTCAACTCGGGCTCAGA	GAACTGCTGCGGGTAGGA	370	chr15:72,006,339–72,006,708
Exon1d	CTCCTACCCGCAGCAGTTC	GGTACTCGGGCAGCTCTTC	227	chr15:72,006,690–72,006,916
Exon1e	CGACCAGGGTTTCGTGTACT	AGGTAGGGCGGCTCCAG	402	chr15:72,006,771–72,007,172
Exon1f	AGCAGGCCTACCCTGACC	GCCTCCAGGAAGTTCTAAGGA	340	chr15:72,007,037–72,007,376
Exon2	CCAACCTGATGCTCTCAATG	CACAGCTAGGCTGGGTTCTG	248	chr15:72,022,182–72,022,429
Exon3	CATGCTGGGTTCTGGTGTC	GAGCTCAGGCACCAAGGTC	271	chr15:72,025,749–72,026,019
Exon4	CAGGGAAGACTAGGCCCTCT	CTGTGAGCAGAGCTGAGTGG	324	chr15:72,026,413–72,026,736
Exon5	CCAGAAACTCCTGAAGGTGG	GGGACATTGGACATGAACATC	232	chr15:72,027,121–72,027,352
Exon6	TTACCACCTTCTCTGGTGAGC	TCCCCAGGCAGGAAAGG	248	chr15:72,028,773–72,029,020
Exon7	CCCTCATTGACCCACTGTCT	GCATGCAGAGCCACAGAGTA	356	chr15:72,031,193–72,031,548

The asterisk indicates the covered genomic regions were based on the March 2006 human reference sequence (NCBI Build 36.1).

### Statistical analysis

Hardy–Weinberg equilibrium (HWE) was examined separately among cases and control subjects using the exact test. The Fisher’s exact test was used to test the allelic association of the SNPs with XFG and POAG. The Bonferroni correction was used to adjust for multiple testing. Taking into account 15 independent tests, the corrected significance threshold was 0.003.

## Results

Fifty XFG patients, fifty POAG patients, and fifty control individuals were recruited into this study. All XFG patients, POAG patients and controls were self-identified as black South Africans speaking a range of southern Bantu languages, including Pedi, Sotho, Tswana, Venda, Xhosa, Zulu, Swazi, Ndebele, and Tsonga. A summary of the clinical phenotypes are recorded in Table 2.

**Table 2:** Demographic and clinical features of study subjects.

**Clinical feature**	**XFG subjects n=50**	**POAG subjects n=50**	**Control subjects n=50**
Age at recruitment (years–mean±SD)	70.4±8.6	57.0±10.1	68.4±9.6
Age at diagnosis (years–mean±SD)	68.0±9.0	54.5±10.6	N/A
Sex			
- Female	20	27	20
- Male	30	23	30
Tribal affiliation			
- Pedi	1	10	5
- Sotho	9	6	6
- Tswana	11	4	11
- Venda	3	1	2
- Xhosa	7	4	3
- Zulu	12	24	14
- Swazi	2	1	2
- Ndebele	2	0	1
- Tsonga	3	0	6
IOP at diagnosis (mmHg–mean±SD)	30.6±12.9	33.3±9.4	13.4±2.5
Cup-disc ratio at diagnosis (mean±SD)	0.8±0.2	0.9±0.1	0.4±0.1

**Figure 1:**
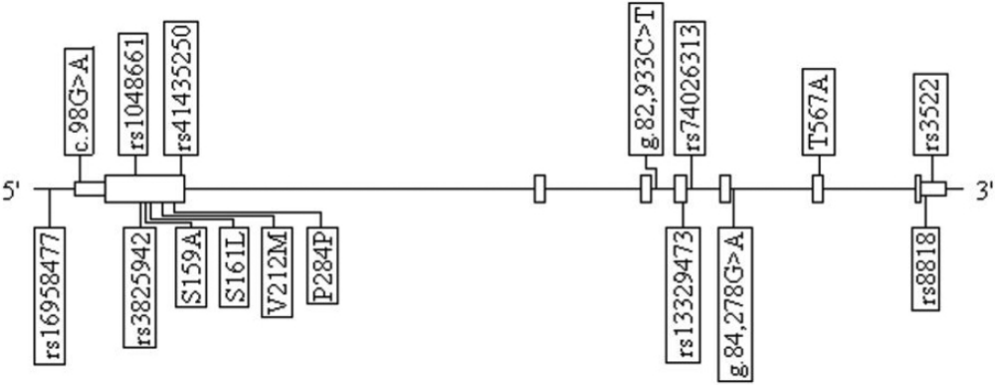
Identified *LOXL1* sequence variants in black South African population. Known SNPs are identified by their rs number designation. Novel coding variants reported as resulting amino acid change. Novel non-coding variants reported as base pair change. Sequence positions are based upon GenBank accession number BC015090 (coding) and AC108137 (genomic). In the image, boxes indicate exons and straight lines indicate introns.

All DNA samples were sequenced using Sanger’s method. Identified *LOXL1* sequence variants are listed in Table 3 for XFG and control individuals. The relative positions of the identified variants are shown in Figure 1. All SNPs were in HWE (p>0.01) in the control group. The most significant association was identified between the common coding changes rs1048661 and rs3825942 and XFG (p=1.7×10^−5^ and p=5.2×10^−13^, respectively), as shown in Table 4. Importantly, the risk allele for rs3825942 was not the G allele described in other populations, but the A allele. While rs74026313, located in the fourth intron, was also associated with XFG (p=2.8×10^−4^). These risk variants remained significant after correction for multiple testing (p≤0.003). Another coding change, rs3522 located in exon 7 was nominally associated with XFG (p=0.02) but did not survive correction for multiple testing. No association with rs16958477 was found (p=0.08; Table 3). This sequence change is located in the promoter region of *LOXL1* and has been reported to be associated with XFG.

**Table 3:** List of coding variants identified from *LOXL1* exon sequencing in South African black individuals with or without exfoliation glaucoma.

**Location**	Nucleotide sequence change*	**Amino acid change**	**SNP ID**	**Allele**	**Allele frequency controls (N)**	**Allele frequency XFG (N)**	**p value**
Promoter	g.62,481A>C	-	rs16958477	A	0.862 (47)	0.947 (47)	0.08
Exon 1	c.98G>A	-	Novel	G	0.990 (48)	1.000 (48)	1.00
Exon 1	c.727G>T	R141L	rs1048661	G	0.810 (50)	0.990 (50)	1.7×10^−5‡^
Exon 1	c.763G>A	G153D	rs3825942	G	0.620 (50)	0.130 (50)	5.2×10^−13‡^
Exon 1	c.780T>G	S159A	Novel	T	0.920 (50)	0.980 (50)	0.10
Exon 1	c.787C>T	S161L	Novel	C	0.970 (50)	1.000 (50)	0.25
Exon1	c.939G>A	V212M	Novel	G	0.990 (50)	0.980 (50)	1.00
Exon 1	c.1,157C>T	P284P	Novel	C	0.990 (48)	1.000 (48)	1.00
Exon 1	c.1,265G>T	A320A	rs41435250	G	1.000 (50)	0.990 (49)	0.49
Intron 3	g.82,933C>T	-	Novel	C	0.917 (48)	0.963 (41)	0.23
Exon 4	c.1,772C>T	F489F	rs13329473	C	0.970 (50)	0.980 (50)	1.00
Intron 4	g.83,628G>A	-	rs74026313	G	0.730 (50)	0.470 (50)	0.00028^‡^
Intron 5	g.84,278G>A	-	Novel	G	0.990 (48)	1.000 (48)	1.00
Exon 6	c.2,004A>G	T567A	Novel	A	0.989 (47)	1.000 (48)	0.50
Exon 7	c.2,130G>C	-	rs8818	G	0.543 (47)	0.521 (48)	0.77
Exon 7	c.2,196C>T	-	rs3522	T	0.674 (43)	0.830 (47)	0.023

The asterisk indicates that nucleotides are numbered as in GenBank accession number BC015090 (coding) and AC108137 (genomic). The double dagger indicates significant after correction for multiple testing.

**Table 4:** Genetic association of common *LOXL1* coding changes with exfoliation glaucoma (XFG) in the black South Africa population.

** **	rs1048661 ** G allele**			rs3825942 ** G allele**		
**Group**	**Frequency**	p value*	OR**	**Frequency**	p value*	OR**
Control	0.810			0.620		
XFG	0.990	1.7×10^−5^	23.2 (3.0–177.2)	0.130	5.2×10^−13^	0.092 (0.045–0.19)

The asterisk indicates Fisher’s exact test comparing to controls. The double asterisk indicates Odds ratio (95% confidence interval).

The functional impact of the two novel, but rare, exon 1 non-synonymous variants S159A and S161L were assessed in silico using PolyPhen and predicted to be benign (PSIC score difference 0.079) and possibly damaging (PSIC score difference 1.685), respectively.

Several coding variants including rs1048661 and rs3825942 were also identified in South African black POAG patients, as listed in Table 5. No association was found between any *LOXL1* sequence variants and POAG (p>0.05, Fisher exact test). The allele frequencies for the G allele of rs1048661 was 0.87 compared to 0.81 for controls (p=0.34) and for the G allele of rs3825942 was 0.60 compared to 0.58 for controls (p=0.88).

**Table 5:** List of coding variants identified from *LOXL1* exon sequencing in South African black individuals with primary open-angle glaucoma.

**Location**	Nucleotide sequence change*	**Amino acid change**	**SNP ID**	**Allele**	**Allele Frequency Controls (N)**	**Allele Frequency POAG (N)**	**p value**
Exon 1	c.727G>T	R141L	rs1048661	G	0.810 (50)	0.870 (50)	0.34
Exon 1	c.763G>A	G153D	rs3825942	G	0.620 (50)	0.600 (50)	0.88
Exon 1	c.780T>G	S159A	Novel	T	0.920 (50)	0.920 (50)	1.00
Exon 1	c.787C>T	S161L	Novel	C	0.970 (50)	0.990 (50)	0.62
Exon 1	c.1,265G>T	A320A	rs41435250	G	1.000 (50)	0.966 (44)	0.10
Intron 3	g.82,933C>T	-	Novel	C	0.917 (48)	0.880 (46)	0.47
Exon 4	c.1,772C>T	F489F	rs13329473	C	0.970 (50)	0.980 (50)	1.00
Intron 4	g.83,628G>A	-	rs74026313	G	0.730 (50)	0.740 (50)	1.00
Intron 5	g.84,278G>A	-	Novel	G	0.990 (48)	0.979 (48)	1.00
Exon 6	c.2,004A>G	T567A	Novel	A	0.989 (47)	0.979 (48)	1.00
Exon 7	c.2,130G>C	-	rs8818	G	0.543 (47)	0.553 (48)	1.00
Exon 7	c.2,196C>T	-	rs3522	T	0.674 (43)	0.620 (47)	0.53

The asterisk indicates that nucleotides are numbered as in GenBank accession number BC015090 (coding) and AC108137 (genomic).

## Discussion

*LOXL1* is one of the lysyl oxidase group of enzymes consisting of *LOX* and the *LOX-* like enzymes 1 to 4. As a group these enzymes are involved in the first step of the formation of cross-links in collagen and elastin. LOXL1 binds to fibulin 5 and tropoelastin at sites of elastogenesis to catalyze the cross-linking that forms elastin polymers [39]. Exon 1 of the *LOXL1* gene encodes the unique NH_2_-terminal domain that is required both for proper enzyme activation and for substrate recognition and binding. It has been postulated that genetic variations in *LOXL1* in this region may contribute to the formation of the pathological fibrillar aggregates accumulating in tissues of patients with XFG [40].

Thorleifsson and coworkers [15] identified significant associations of XFS/XFG with the non-synonymous coding changes rs1048661 and rs3825942 in the Caucasian population. It has been hypothesized that one or both of these SNPs are causally involved in the pathobiology of XFS although no evidence beyond genetic association has been reported to date. However, several recent observations now argue against this hypothesis. Multiple studies reported that rs1048661 was not associated with XFS/XFG in all populations (Table 6) [32-34]. Furthermore, although the G allele of rs1048661 was associated with an increased XFS/XFG risk in Caucasian populations, the opposite (T) allele was associated with an increased risk in the Chinese and Japanese populations (Table 6) [25-31]. Our finding for rs3825942 in the South African black population mirrors this previously reported result for rs1048661. Further evidence against a causal role of rs3825942 in XFG stems from reports that this SNP does not appear to affect *LOXL1* gene expression levels in blood or ocular tissues [15,41] and that it may represent a conservative substitution based on the in silico prediction programs Polymorphism Phenotyping (PolyPhen) and Sorting Intolerant From Tolerant (SIFT) [34]. Taken together, these data suggest that other functional risk factors in *LOXL1* remain to be identified.

**Table 6:** Summary of the genetic association of two coding variants in *LOXL1* gene with XFS/XFG.

** **	rs1048661 ** G allele**		** **	rs3825942 ** G allele**		** **	** **
**Studied population**	**Case**	**Control**	**Significant association**	**Case**	**Control**	**Significant association**	**Reference**
Icelandian	0.781	0.651	Yes	0.984	0.847	Yes	[15]
Swedish	0.834	0.682	Yes	0.995	0.879	Yes	[15]
American	0.819	0.600	Yes	0.986	0.880	Yes	[16]
Australian	0.78	0.660	Yes	0.95	0.84	Yes	[17]
American	0.787	0.665	Yes	0.939	0.844	Yes	[18]
American	NA	NA	NA	1.000	0.856	Yes	[19]
American	0.843	0.703	Yes	0.959	0.798	Yes	[20]
Austrian	0.841	0.671	Yes	0.994	0.817	Yes	[21]
Germany	0.818	0.644	Yes	0.951	0.857	Yes	[22]
Italian	0.825	0.693	Yes	1.000	0.821	Yes	[22]
Finnish	0.825	0.683	Yes	0.968	0.823	Yes	[23]
Germany	0.844	0.660	Yes	0.992	0.856	Yes	[24]
Chinese	0.110	0.480	Yes	1.000	0.900	Yes	[25]
Japanese	0.036	0.493	Yes	1.000	0.877	Yes	[26]
Japanese	0.008	0.460	Yes	1.000	0.857	Yes	[27]
Japanese	0.006	0.450	Yes	0.994	0.853	Yes	[28]
Japanese	0.005	0.474	Yes	0.995	0.850	Yes	[29]
Japanese	0.005	0.497	Yes	0.986	0.863	Yes	[30]
Japanese	0.005	0.554	Yes	0.993	0.806	Yes	[31]
Indian	0.721	0.634	No	0.923	0.742	Yes	[32]
Chinese	0.542	0.444	No	0.992	0.918	Yes	[33]
American	0.829	0.719	No	0.988	0.795	Yes	[34]
South African	0.990	0.810	Yes	0.130	0.620	Yes	This study

In the table, NA indicates not available; *LOXL1* indicates lysyl oxidase-like 1; XFS indicates exfoliation syndrome; and XFG indicates exfoliation glaucoma.

Interestingly, XFS and XFG are rarely found in the African American and West African populations [10,42]. Why XFS is so rare in persons of West African descent is puzzling, especially in light of a recent report confirming the presence of major *LOXL1* risk variants in these populations [43]. The low XFS/XFG prevalence suggests that other genetic or environmental factors, and not just *LOXL1*, contribute to the pathogenesis of this condition. This is the first association study of *LOXL1* in XFG patients performed in an ancestral African population. We have confirmed the association of the major non-synonymous coding variants rs1048661 and rs3825942 with XFS/XFG. However, in the black South African population, the risk at rs3825942 is the A allele not the G allele observed to increase risk in all other reported populations. Interestingly, in contrast to non-African populations with XFG, for whom the major (more common) allele confers an increased risk, the allele is the minor allele in this population. This finding suggests that none of the currently known sequence variants in *LOXL1* may be critical for the exfoliation phenotype.

In summary, this study corroborates the genetic association of *LOXL1* sequence variants with XFG in a South African black population and has generated two novel results. First, rs3825942 (G153D) risk allele is reversed in this population compared with non-African populations suggesting that this SNP may only be a proxy for the as yet unknown causal variant(s) in *LOXL1*. This finding is similar to previously reported results for rs1048661 in Asian populations. Second, we did not observe an XFG association with SNP rs16958477, which was hypothesized to affect the *LOXL1* promoter activity. In light of our study and the work reported by others, further analysis of the promoter and other regulatory regions of the *LOXL1* gene in the Caucasian, Asian, and African populations is warranted.
